# Improvement of the suture-occluded method in rat models of focal cerebral ischemia-reperfusion

**DOI:** 10.3892/etm.2014.1483

**Published:** 2014-01-10

**Authors:** PING ZHANG, ZHEN HUANG, HAI-QING YAN, LIN-LIN SU, YONG-KUN GUI, HAI-XIA LV, BIN ZHU, TONG LI

**Affiliations:** 1 Department of Neurology, the First Affiliated Hospital of Xinxiang Medical University, Xinxiang, Henan 453100, P.R. China; 2 Department of Pharmacy, Xinxiang Medical University, Xinxiang, Henan 453003, P.R. China

**Keywords:** rat, focal cerebral ischemia-reperfusion, model

## Abstract

The aim of the present study was to provide a simple method of establishing a rat model for focal cerebral ischemia-reperfusion (FCIR). The suture-occluded method was used to establish FCIR in male Sprague-Dawley rats. An incision was made over the bifurcation of the common carotid artery (CCA), through which a suture was inserted up to the internal carotid artery (ICA). The suture remained in the skin subsequent to model establishment and was withdrawn to the CCA to enable reperfusion. The reliability of the rat model was assessed via analysis of nerve function, tetrazolium (TTC) staining and pathological examination. Following FCIR in rats, the resulting neurological impairments were observed. TTC staining revealed infarcts and pathological examination revealed typical pathological changes. This modified method was simple, reliable and, therefore, may be used to investigate FCIR.

## Introduction

Cerebral ischemic stroke has a high incidence of morbidity and mortality and results in a severe disability with a complex pathogenesis, which is unclear. Thus, this condition threatens the safety and quality of life of humans. Although the treatment for cerebral ischemic stroke has significantly improved recently, none of the available drugs for its treatment and prevention are considered to be optimal. The establishment of an optimal animal model, which simulates human cerebral ischemia may allow future investigations into the pathogenesis of, and prevention measures for, cerebral ischemia in humans.

Rats are selected as experimental animals due to their similarities to humans with regard to cerebrovascular anatomy and function. Furthermore, rats are of a standard species or strain, exhibit good intraspecific homozygosity and experimental repeatability and routine monitoring can be conveniently performed. There are numerous factors that influence the selection of rats as models; for example, oestrogen protects female rats from cerebral injury, therefore, male rats are included in investigations (1). Elderly rats are excluded due to the anatomical and pathological changes, which occur in their middle cerebral artery (MCA) and common carotid artery (CCA), such as tortuosity, luminal stenosis and arteriosclerosis (2). Furthermore, model establishment using adult rats leads to a relatively high success rate (3). Moreover, certain species of rats affect the model; for example, a small variation in the MCA of Fischer-344 rats results in a good consistency of cerebral infarct volume in the MCA occlusion (MCAO) model, whereas Wistar-Kyoto rats exhibit the smallest cerebral infarct volume and exhibit the largest variation. Characteristics of Sprague-Dawley (SD) rats fall between the abovementioned rat species; therefore, Fischer-344 rats are optimal for model establishment, however, they are rarely used as they are difficult to obtain. SD rats differ marginally from Wistar rats; however, intraoperative bleeding is minimal in SD rats, which makes surgery less complex. SD rats grow at a faster rate, are milder and less expensive; thus, they are often selected as experimental animals (4).

MCAO is the predominant cause of cerebral ischemic stroke (5) and the suture-occluded method is extensively used to establish MCAO models for performing clinical research (6). The conventional suture-occluded method established by Longa *et al* (7) is the prevailing method for the focal cerebral infarction model. However, it is complicated, time consuming and inevitably results in ischemic infarction of tissues (such as the temporalis, lingualis and pharyngeal muscles), to which the external carotid artery (ECA) supplies blood, which leads to further complications. To provide an optimal, reliable and simple animal model for FCIR injury research, a modified suture-occluded method of Longa *et al* was used. The experience of establishing a model using a modified process through anatomical observation of rats and an analysis of the key steps in establishing the model was also summarised.

## Materials and methods

### Animals

In total, 24 healthy, male SD rats (weight, ~220–260 g) were obtained from the Laboratory Animal Center of Southern Medical University (Guangdong, China) and were randomised into the sham-surgery (n=12) and surgery (n=12) groups. In each group, six rats were used for tetrazolium (TTC) staining and six to obtain tissue sections. The present study was conducted in strict accordance with the recommendations in the Guide for the Care and Use of Laboratory Animals of the National Institutes of Health. The animal use protocol was reviewed and approved by the Institutional Animal Care and Use Committee of the First Affiliated Hospital of Xinxiang Medical University (Henan, China).

### Suture preparation

Nylon sutures (diameter, ~0.2–0.25 mm) were cut to a 60 mm length. One end of the suture was heated to form a smooth, spherical shape (diameter, ~0.26–0.3 mm), which was observed under a BH2 microscope (Olympus Corporation, Tokyo, Japan); the texture of the opposite end was rough. The suture was labelled with the required length, sterilised with alcohol and stored in heparin saline solution.

### Establishment of the experimental model

The rats were fasted for 24 h prior to the experimental procedures, anaesthetised intraperitoneally using 10% chloral hydrate (3 ml/kg), fixed in a supine position and incised at the midline of the neck, (incision length, 20 mm). The left CCA, internal carotid artery (ICA) and external CA (ECA) were exposed. The proximal CCA was ligated and the suture was suspended around the distal CCA for subsequent use, then the ECA was ligated at the bifurcation of the CCA. The proximal ICA was tied with a slipknot and the distal ICA was clamped using an artery clamp. A nylon suture was introduced into the ICA lumen through a puncture wound from a micro-incision, which was 5-mm distal to the CCA bifurcation. The slipknot was tightened and the artery clamp was removed. Insertion ceased once ~19 mm of nylon suture had been inserted or until resistance was felt (where the tip of the suture reached the origin of the MCA), which resulted in the occlusion. The prepared suture was tied and fixed with a subcutaneously embedded thread residue; subsequently, the incision was closed. After 2 h, the rats were anaesthetised with diethyl ether, all the occlusions were removed (2 needles) and the 10 mm nylon suture was withdrawn gently to reperfuse blood flow. In the sham-surgery group, 10 mm of nylon suture was inserted into the ICA lumen, and the remaining procedures were performed as described above. The intraoperative room temperature was maintained at ~20–30°C and the postoperative anal temperature of the rats was 37±0.5°C.

### Neural function deficit score (NFDS)

The neurological status of the rats was assessed 24 h following ischemia-reperfusion (I/R) according to the method described by Longa *et al* (7). The rats were classified into five grades: Grade 0, no neurological impairment; grade 1, mild neurological impairment, failure to fully stretch the contralateral forelimb; grade 2, moderate neurological impairment, rotation to the contralateral side; grade 3, severe focal neurological impairment, toppling to the contralateral side; and grade 4, unable to walk spontaneously, exhibiting conscious disturbance.

Tetrazolium (*TTC) staining.* The rats were intraperitoneally overdosed with 10% chloral hydrate and were sacrificed by decapitation 72 h following I/R. Their brains were immediately harvested and frozen at −20°C. After 20 min, five coronal sections (2 μm) were incised from the upper forehead using a tissue slicer and incubated in TCC staining solution at 37°C for 30 min. The sections were then fixed in fresh 10% formaldehyde for ≥24 h.

### Nissl staining

The rats were intraperitoneally overdosed with 10% chloral hydrate and sacrificed by decapitation 72 h after I/R. The chests were opened and the left ventricle (LV) was intubated; the blood was flushed with 4°C normal saline, followed by fixation of the LV with 4% paraformaldehyde phosphate buffer, overnight, at 4°C. The LV was stored in 20–30% sucrose phosphate buffer at 4°C until the tissue sank to the bottom of the solution. The temporal lobe was frozen and serially cut. The section was stained in Toluidine blue (Wuhan Boster Biotechnology Company, Wuhan, China) at 37°C for 5 min, washed in distilled water for 3 min. Conventional dehydration was then conducted using gradient alcohol and the lobe sections were mounted using a neutral balsam.

## Results

### Neurobehavioral observation

**Figure 1:**
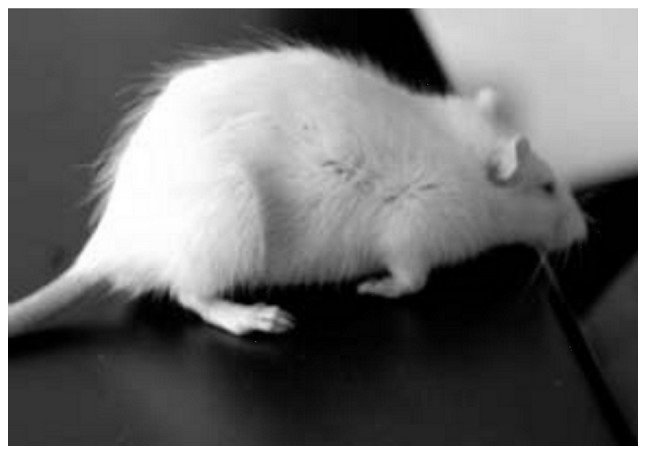
The rat exhibited an infarction in the left middle cerebral artery, with right limb paralysis and rotation to the contralateral side.

**Figure 2:**
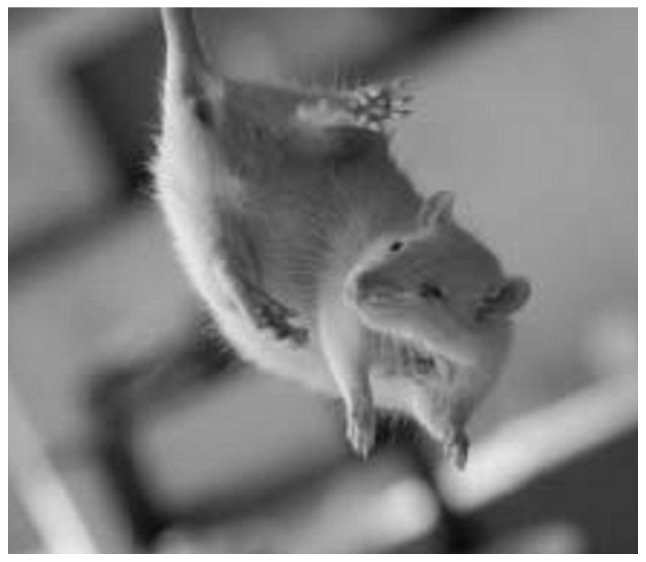
The rat exhibited an infarction in the left middle cerebral artery and the right upper limb and impairment of muscle strength was apparent.

**Figure 3:**
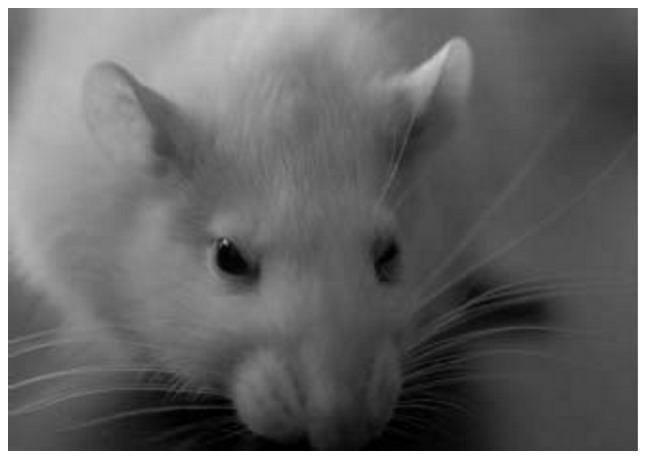
The rat exhibited a decreased palpebral fissure and corestenoma on the left side.

In the sham-surgery group, no neurobehavioral abnormality of the contralateral limb was observed: the rats were active and walked and drank well. Neurobehavioral impairments were evident in the surgery group: i) The rats exhibited hydroadipsia and paralysis of the contralateral limb, turned or rotated to the contralateral side, limped, and appeared dispirited and inactive (Fig. 1). ii) The results of the tail suspension test were positive, the rats failed to bend their contralateral forelimb when their tails were held, or were not able to independently move their contralateral limbs, and demonstrated spontaneous rotation to the contralateral side (Fig. 2). iii) The rats exhibited a decreased ipsilateral palpebral fissure and corestenoma (Fig. 3). A score of 1–3 indicated that the model had passed the test.

### TTC staining

**Figure 4:**
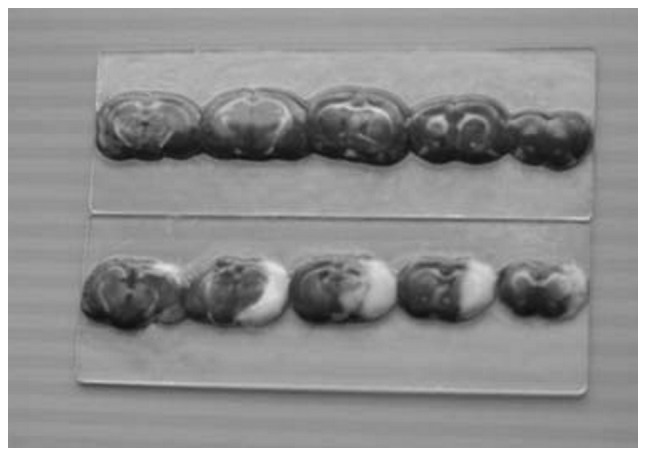
The surgery group exhibited focal cerebral ischemia in the parietal cortex, with a morphology change in the hippocampal neuron. The left middle cerebral artery presented as white.

In the sham-surgery group, no infarcts were observed 72 h following I/R, whereas the surgery group demonstrated large areas of infarct, which were predominantly located in the parietal cortex and lateral striatum, or at the hippocampus and cerebral hemisphere. The left middle cerebral artery appeared white in colour, indicating that there was no haemorrhaging (Fig. 4). Furthermore, no apparent haemorrhaging was observed in the infarcts.

### Nissl staining

**Figure 5:**
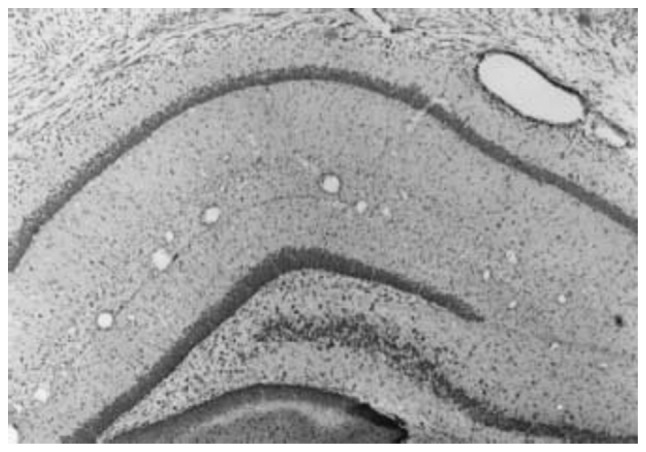
Nissl staining in the sham-surgery group. The neurons in the cerebral cortex and hippocampus were densely distributed (magnification, ×100).

**Figure 6:**
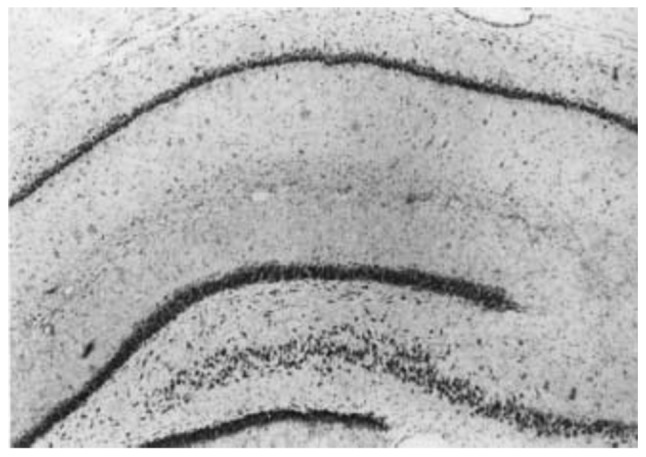
Nissl staining in the surgery group. The neuronal density of the cerebral cortex and hippocampus was decreased and the pyramidal cell layer was thinned (magnification, ×100).

The Nissl-stained histological sections were observed under a BH2 microscope. In the sham-surgery group, each layer of neurons in the cerebral cortex was densely arranged, specifically in layers II to VI. Occasional neuron distribution in layer I was observed. In layer V, the pyramidal cells were closely arranged and had larger cell bodies. A dense distribution of pyramidal cells was observed in the hippocampal CA1 region, which was divided into 2–5 layers. The cell contour was clear, with root-like apical dendrites that were apparent towards the radial layer (Fig. 5). In the surgery group, the ischemic injury involved the cerebral cortex, hippocampus, dentate gyrus and additional brain regions. The cerebral cortex presented six layers and the neurons were predominantly distributed in layers II to IV and exhibited a decreased density. The number of pyramidal cells in layer V was markedly decreased. In the hippocampal CA1 region, the pyramidal cell layer was thin, with a sparse distribution (Fig. 6).

**Figure 7:**
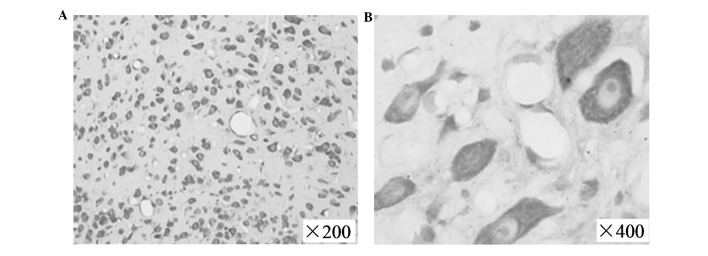
Nissl staining in the sham-surgery group, with the neurons in the ischemia cortex exhibited intact morphologies. (A) The spindle-shaped and (B) granular Nissl bodies were evenly distributed.

**Figure 8:**
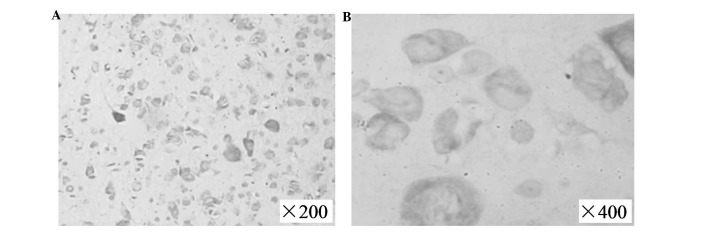
Nissl staining in the surgery group, 72 h following ischemia-reperfusion. (A) The Nissl bodies revealed a diffuse disappearance with residue in the individual cells. (B) The neurons were lost and cell ruptures were observed.

In the sham-surgery group, the neurons in the cerebral cortex demonstrated intact morphologies. The Nissl bodies appeared purple-blue in color, whereas the nucleus was light blue. The cells were plump, with evenly distributed Nissl bodies observed in the cytoplasm (Fig. 7). In the surgery group, the neurons in the cerebral cortex demonstrated ischemic changes, including decreased neuron density, shortened apical dendrites, swollen cells (which appeared to be ruptured), broadened intercellular spaces and neuron disintegration (Fig. 8).

## Discussion

Ischemic cerebrovascular disease is a predominant human cerebrovascular disease and >60% of cases frequently occur in the MCA. The cerebrovascular structure of rats is similar to that of humans in that the ICA and vertebrobasilar system form the Circle of Willis, which supplies blood to the brain through the arterial branches. Thus, rats are recognised as the optimal animal for establishing a cerebral ischemic model. The model is divided into global and focal cerebral I/R, according to the ischemic scope, or persistent and transient ischemia, according to the ischemia time. Among all of the animal models pertaining to strokes, the predominant model is MCAO, which is induced by focal cerebral ischemia in rats (8). MCAO can result in dysneuria, such as, typical limb hemiplegia and cerebral infarcts, which facilitate the observation and assessment of cerebral ischemia. Various methods of establishing a rat model of ischemia have been proposed including electrocoagulation, the suture-occluded method, cerebral embolisation, photochemically induced thrombosis and mechanical occlusion with craniectomy. The suture-occluded method is the commonly used approach to establish a cerebral infarction rat model (9–12), as demonstrated by Koizumi *et al* (10) in 1986 and improved by Longa *et al* in 1989 (7). The suture-occluded method without craniotomy is specifically suitable for investigations into FCIR injury due to the minor trauma area and the constant ischemic area (13). However, Longa’s method requires separation and ligation of the ECA and the extracranial branch of the ICA-pterygopalatine artery as well as insertion of the suture into the ECA, which is complex to perform in rats due to the small surgical field, difficulty of separation, tendency for bleeding and the involvement of microinstruments; thus, its application is limited.

The suture-occluded method of Zea-Longa (7) was used in the present study to establish the FCIR model, with certain modifications: i) Incisions were made at the bifurcation of CCA to insert the suture from the CCA to the ICA, which avoided the insertion into the ECA and bypassed the bend at the CCA bifurcation; thus, suturing was straightforward. ii) Cerebral infarction severity was not affected by ligation of the pterygopalatine artery. The suture was mistakenly inserted into the pterygopalatine artery, however, the resistance was felt after inserting 10 mm of suture; thus, it was withdrawn and adjusted to a different angle, which was consistent with the study (8). iii) The suture was subcutaneously embedded following establishment of the models; reperfusion was conducted under rapid anaesthesia by unpicking one to two stitches, withdrawing the suture to the CCA, cutting the suture near to the skin and closing the incision. This generally avoided vessel injury, on removal of the suture, as well as movement and slippage of the suture that resulted from movement of the rat following revival, which enhanced the success rate and reliability of the model. iv) Additional surgery did not influence the healing; rapid anaesthesia with diethyl ether enabled the reperfusion process to be completed in 5 min without altering the experimental conditions, which ensured homogeneity of the model. However, there were numerous factors that affected the model: the surgical process was standardised as it was performed at the same time each day, the rats were randomised into different groups, the age, gender and body weight of the rats was consistent, in addition to maintaining a constant diameter of the nylon suture and the spherical shape at the ends.

*In vivo* evaluation of a successful rat model includes monitoring the MCA blood flow and electroencephalograph, distinction between cortical and subcortical infarction by positron emission tomography and magnetic resonance imaging (MRI), detection of the cerebral ischemic core and determination of the ischemic penumbra (14,15). Instrument monitoring is accurate, however, it is expensive and limited by the experimental conditions; specifically when a large quantity of animals are investigated. An optimal NFDS system should be non-invasive, low cost, reliable, time saving and straightforward to perform. The symptoms of focal cerebral ischemia are characterised by contralateral limb dysfunction; therefore, the neuropathy symptom score is internationally regarded as a general criteria. Numerous scoring methods exist including, the Bederson method (16), the Nagaoka method (17), the Longa method (7), the Tatlisumak method (18) as well as the Hattori method (19). Previous studies have confirmed that the NFDS is able to evaluate MCA infarction due to the close association between them, moreover, behavioural scores have been demonstrated to correlate with the cerebral infarct volume (20). During the establishment of the MCAO model using numerous SD rats, Boyko *et al* (21) identified the association of the behavioural score with the infarct size and demonstrated the correlation between the predicted and actual values using MRI. Neurobehavioral analysis, independent of any devices, is a valuable, effective, reliable and sensitive method of evaluating the nervous system. Numerous scholars have suggested increasingly comprehensive evaluation methods, including the scoring of neurological and vestibular function in ischemic animals, which results in an improved assessment. The NFDS by Longa *et al,* was used in the present study and the model was labelled as successful if the rats accorded with any indicators of the scoring system. However, ipsilateral Horner’s syndrome resulted from the intraoperative injury of the superior cervical sympathetic nerve, which resulted in a decreased palpebral fissure and corestenoma and, therefore, failed to serve as an individual sign of a successful model.

The surgical principle of the FCIR model was: The suture (length, 19 mm) in the ICA simultaneously blocked the two sources of arterial blood in the Circle of Willis; the ipsilateral ICA and the posterior communicating artery that connects with the vertebral artery, while the ipsilateral anterior CA maintained the ability to obtain blood from the contralateral ICA. On withdrawal of the suture to the ICA, the ischemic area was reperfused through the contralateral ICA and vertebrobasilar artery of the Circle of Willis. The ischemia time was ~2–6 h in the rat cerebral I/R injury model. The interaction between the four physiopathologic mechanisms, including toxicity of excitatory amino acids, peri-infarct depolarisation, inflammation and cell apoptosis following I/R (22), further aggravating the symptoms of ischemia and reaches a steady state within ~3–6 h. Kawamura *et al* (23) demonstrated that ischemia time, which was >3 h, resulted in the reperfusion rats exhibiting comparable neurologic manifestations, cerebral infarct volume and encephaledema to those rats without reperfusion following 24 h of ischemia. Ischemia duration <1 h, led to reperfusion resulting in reduced infarcts and greater variation, an ischemia duration of 2 h falls between the abovementioned conditions. Therefore, reperfusion was performed following an ischemia duration of 2 h. In addition, cerebral edema peaked three days after the clinical cerebral infarction; thus, TTC staining was conducted on the third day.

In conclusion, the results of the present study revealed that the modified rat MCAO model exhibited evident indicators of neurologic deficit and resulted in constant infarct locations. The sham-surgery group demonstrated integral brain structure without ischemic variation, and no infarcts were observed following TTC staining, which further demonstrated the reliability of the model.
